# Metformin Reduces Lipotoxicity-Induced Meta-Inflammation in *β*-Cells through the Activation of GPR40-PLC-IP3 Pathway

**DOI:** 10.1155/2019/7602427

**Published:** 2019-12-18

**Authors:** Ximei Shen, Beibei Fan, Xin Hu, Liufen Luo, Yuanli Yan, Liyong Yang

**Affiliations:** ^1^Endocrinology Department, The First Affiliated Hospital of Fujian Medical University, Fuzhou, 350005 Fujian, China; ^2^Diabetes Research Institute of Fujian Province, Fuzhou, 350005 Fujian, China

## Abstract

**Background and Purpose:**

Metformin, a widely used antidiabetic drug, has been shown to have anti-inflammatory properties; nevertheless, its influence on *β*-cell meta-inflammation remains unclear. The following study investigated the effects of metformin on meta-inflammatory in *β*-cells and whether the underlying mechanisms were associated with the G protein-coupled receptor 40-phospholipase C-inositol 1, 4, 5-trisphosphate (GPR40-PLC-IP3) pathway.

**Materials and Methods:**

Lipotoxicity-induced *β*-cells and the high-fat diet-induced obese rat model were used in the study.

**Results:**

Metformin-reduced lipotoxicity-induced *β*-cell meta-inflammatory injury was associated with the expression of GPR40. GPR40 was involved in metformin reversing metabolic inflammation key marker TLR4 activation-mediated *β*-cell injury. Furthermore, downstream signaling protein PLC-IP3 of GPR40 was involved in the protective effect of metformin on meta-inflammation, and the above process of metformin was partially regulated by AMPK activity. In addition, the anti-inflammatory effects of metformin were observed in obese rats.

**Conclusion:**

Metformin can reduce lipotoxicity-induced meta-inflammation in *β*-cells through the regulation of the GPR40-PLC-IP3 pathway and partially via the regulation of AMPK activity.

## 1. Introduction

Metformin has been widely used for the treatment of type 2 diabetes. Metformin exerts its effects through AMPK activation. Increasing evidence indicates that metformin directly acts on pancreatic *β*-cells [1]; nevertheless, the effect of metformin on *β*-cells remains controversial. Studies have shown that metformin inhibits *β*-cell apoptosis induced by hyperglycemia or hyperlipidemia [2, 3] and enhances the number of pancreatic progenitor cells in the mouse embryonic pancreas [4], while other studies have indicated that metformin has a dual role in MIN6 pancreatic *β*-cell function through the AMPK-dependent autophagy pathway [5]. Therefore, the exact regulation mechanisms of metformin on *β*-cells need to be further investigated.

Metabolic inflammatory injury is currently considered an important mechanism for lipotoxicity-induced *β*-cell injury. However, targeted interventions have not yet been identified. Toll-like receptor 4 (TLR4) is the key marker of meta-inflammation which has an important role in the pathogenesis of metabolic inflammation in type 2 diabetes [6–10]. Our previous studies have demonstrated that lipotoxicity directly activates the TLR4-JNK pathway cascade in islet *β*-cells and that it induces insulin secretion disorders and apoptosis of *β*-cells [11]. Whether the protective effect of metformin on *β*-cells is associated with TLR4 has not yet been reported. Studies have suggested that metformin has a clear inhibitory effect on TLR4 in the skeletal muscle of diabetic rats [12]. The aim of this study was to investigate whether metformin could inhibit the activation of TLR4 inflammatory signals in *β*-cells induced by lipotoxicity.

GPR40 is a long-chain FFA receptor that is specifically expressed on the membrane of pancreatic *β*-cells [13]. Although the early literatures reported that GPR40 has inconsistencies in beta cell dysfunction [14–16], many studies supported the benefit of GPR40 on beta cells [17, 18]; and GPR40 agonist was already tested in several clinical trials [19–21]. At present, the association between the protective effects of metformin and GPR40 has not yet been reported. However, preclinical studies have demonstrated that a combination therapy with GPR40 agonist TAK-875 and metformin could be a valuable strategy for glycemic control and *β*-cell preservation in type 2 diabetes [22]. Thus, this study is aimed at further exploring the relationship between GPR40 and the protective effects of metformin on lipotoxicity-induced metabolic inflammation.

In summary, *β*-cell lines and high-fat diet-induced obese rats were employed to investigate the protective effects of metformin on lipotoxicity in *β*-cells and the relationship between this protective effect and GPR40. Furthermore, it was confirmed that metformin inhibits lipotoxicity-induced TLR4 activation through the GPR40 pathway and AMPK activity, thereby preventing lipotoxicity-induced apoptosis of *β*-cells.

## 2. Materials and Methods

### 2.1. Cell Culture

Mouse islet cell line *β*TC6 was obtained from the American Type Culture Collection (Manassas, VA, USA). Cells were incubated with Dulbecco's modified Eagle's medium (DMEM, 25 mmol/L glucose), supplemented with 10% fetal bovine serum (FBS), 2 mmol/L L-glutamine, and 10 mmol/L HEPES buffer solution in a humidified atmosphere containing 5% CO_2_/95% air at 37°C. All reagents were purchased from Gibco (Carlsbad, USA). Cells at passages 26 to 30 were used for all experiments.

### 2.2. In Vitro Cell Experiments

#### 2.2.1. Palmitic Acid (PA) or Lipopolysaccharide (LPS) Treatment

*β*TC6 cells were incubated in DMEM (25 mmol/L glucose) for 24 h until the cells reached 50% confluence. Cell culture medium was then removed and washed twice with PBS then treated with 0.5 mmol/L PA (Sigma-Aldrich) or 1.0 mg/L LPS (Sigma-Aldrich) for 24 h in culture media. Cells treated with 0.5% BSA/ethanol in HDMEM without PA or LPS (uninduced) served as the control. PA and LPS solutions were prepared as described before [11].

#### 2.2.2. Metformin (MF) Treatment

*β*TC6 cells were treated with 0.5 mmol/L PA+DMEM (25 mmol/L glucose) or 1.0 mg/L LPS+DMEM (25 mmol/L glucose) for 24 h and then exposed to different concentrations (25, 50, and 100 *μ*mol/L) of metformin (dissolved in 0.1% dimethylsulfoxide (DMSO) (*v*/*v*); BN1504091105; Chia Tai Tianqing, China) [23] with complete medium for 72 h. PA-induced or LPS-induced cells without metformin and uninduced cells exposed to metformin served as controls.

#### 2.2.3. GPR40 Agonist and Inhibitor Treatment

*β*TC6 cells were pretreated with 1.0 mg/L LPS for 24 h. Afterwards, cells were washed twice with PBS and then treated with 10 *μ*mol/L of TAK-875 (dissolved in 0.1% DMSO (*v*/*v*); TRC-CANADA) [24] or 5 *μ*mol/L of DC260126 (dissolved in 0.1% DMSO (*v*/*v*); TRC-CANADA) [25] for 72 h. LPS-induced cells without TAK-875 or DC260126 and uninduced cells exposed to TAK-875 or DC260126 served as controls.

#### 2.2.4. Protein Inhibitor Treatments

*β*TC6 cells were pretreated with 20 *μ*mol/L PLC inhibitor (U73122, Sigma-Aldrich) [26] or 20 *μ*mol/L IP3 inhibitor (2-APB, Sigma-Aldrich) [27], respectively, for 4 h before being exposed to 0.5 mmol/L PA+DMEM (25 mmol/L glucose)+100 *μ*mol/L MF for 72 h. Cells exposed to U73122, 2-APB, or complete medium alone were used as controls.

#### 2.2.5. AMPK Inhibitor and Agonist Treatment

*β*TC6 cells were pretreated with 10 *μ*mol/L AMPK inhibitor (compound C; Sigma-Aldrich, USA) [28] for 4 h before being exposed to 0.5 mmol/L PA+DMEM (25 mmol/L glucose)+100 *μ*mol/L MF for 72 h. Cells exposed to compound C or complete medium alone were used as controls.

*β*TC6 cells were pretreated with 0.5 mmol/L PA+DMEM (25 mmol/L glucose) for 24 h. Consequently, cells were treated with 1 mmol/L of AMPK agonist AICAR (dissolved in 0.1% DMSO (*v*/*v*); Henan DaKen Chemical CO., LTD.) [29] for additional 72 h. PA-induced cells+DMEM (25 mmol/L glucose) without AICAR and uninduced cells exposed to AICAR served as controls.

### 2.3. GPR40 Small Interfering (si) RNA and GPR40 Cloning and Adenovirus Generation

The specific experimental process was based on our previous methods [24].

### 2.4. Glucose-Stimulated Insulin Secretion (GSIS)

The detection steps were carried out according to our previous methods [11], and the total cellular protein concentration was used to correct the concentration of insulin.

### 2.5. Animal Experiments

Five- to six-week-old, specific-pathogen-free grade, male Sprague-Dawley (SD) rats (220 ± 10 g) were obtained from the laboratory animal facility at Fujian Medical University (animal certification number SYXK (min) 2016-0006). After a one-week acclimation period, the animals were randomly divided into two groups: normal control diet (*n* = 20) and high-fat diet (HFD, *n* = 20). Rats in the control group were additionally divided into two groups, half of which received metformin (50 mg/kg/d; fasting gavage, per day; *n* = 10) [30], while others received no treatment (*n* = 10). All rats in the control group were fed a normal pellet diet for 16 weeks. Rats in the HFD group received a high-fat diet [11] for 16 weeks to induce obesity. The HFD group was then subdivided into two groups, half of which received metformin (50 mg/kg/d; fasting gavage, per day; *n* = 10), while others received no treatment (*n* = 10). The body weight and length were recorded weekly.

This study was approved by the Ethics Committee for Biomedical Research of the First Affiliated Hospital of Fujian Medical University.

### 2.6. ITT and IPGTT

#### 2.6.1. Insulin Tolerance Test (ITT)

All animals were fasted for 4 hours. Insulin was injected at 1 U/kg [31]. Blood glucose was measured before and 0, 30, 60, 90, and 120 min after the injection of insulin.

#### 2.6.2. Intraperitoneal Glucose Tolerance Test (IPGTT)

Intraperitoneal glucose tolerance test was preformed according to a previously described protocol [32]. Briefly, rats were fasted for 8 hours. Then, all animals received an intraperitoneal injection of 50% glucose (2 g/kg). Blood glucose and insulin were measured before and 0, 30, 60, 90, and 120 min after the injection of glucose. After the experiment, the feed was supplemented.

### 2.7. Serum Insulin, FFA, and Biochemical Indicator Measurements

Control and experimental rats were fasted overnight for 8 hours and then euthanized by intraperitoneal injection of 10% chloral hydrate (0.03 mL/kg). Blood samples were obtained from the abdominal aorta (serum tube, without anticoagulant); samples were centrifuged at 3500 rpm for 10 minutes at room temperature and then separated and stored at -80°C. Blood samples were used to measure fasting plasma glucose, fasting plasma insulin, free fatty acid (FFA), triglycerides (TG), total cholesterol (TC), HDL cholesterol (HDL-C), and LDL cholesterol (LDL-C), based on previously published methods [11].

### 2.8. Enzyme-Linked Immunosorbent Assay

#### 2.8.1. Inflammatory Cytokine Measurements

Interleukin-1 (IL-1), interleukin-6 (IL-6), and tumor necrosis factor-*α* (TNF-*α*) were detected by ELISA (Cusabio, China). Intra-assay coefficient of variation was <8% and interassay coefficient of variation was <10%.

#### 2.8.2. Cellular IP3 Measurements

Cellular IP3 detection referenced the ELISA instructions (Cusabio, catalog number CSB-E13420m, http://www.cusabio.com,) and documentary reports by Li et al. [33]. Cells were thoroughly washed with precooled PBS (pH 7.2 to 7.4), then transferred to a suitable centrifuge tube, diluted with 1× PBS (pH 7.2 to 7.4) to a cell concentration of 100 million/mL, and stored at -20°C overnight. Repeat freeze-thaw cycles were used to disrupt the cell membrane. The lysed cells were then centrifuged at 1000 g for 2 min at 2-8°C, and the supernatant was used for analysis. Anti-IP3 detection antibody was added to the supernatant and incubated at 37°C for 1 hour and then incubated with substrate solution at 37°C for 15 min. The reaction was terminated following the addition of stop solution, and the OD value was measured at a wavelength of 450 nm using a microplate reader. Using the standard concentration as the ordinate (logarithmic coordinate) and the corresponding OD value as the abscissa (logarithmic coordinate), the standard curve was drawn using GraphPad Prism 5 software and the concentration for each sample was calculated.

### 2.9. Apoptosis Analysis by TUNEL Assay

#### 2.9.1. Detection of *β*TC6 Cell Apoptosis

The TUNEL was performed using the DeadEnd™ Fluorometric TUNEL System (DeadEnd™ Fluorometric TUNEL System, Promega Corporation, USA), according to the previously published method [11]. Mechanism: the DeadEnd™ Fluorometric TUNEL System measures the fragmented DNA of apoptotic cells by catalytically incorporating fluorescein-12-dUTP at 3′-OH DNA ends using the Terminal Deoxynucleotidyl Transferase, Recombinant, enzyme (rTdT), which forms a polymeric tail using the principle of the TUNEL (TdT-mediated dUTP Nick-End Labeling) assay. The fluorescein-12-dUTP-labeled DNA then can be visualized by fluorescence microscopy or quantified by flow cytometry. In addition, the nucleus is stained with red fluorescence by PI (https://www.promega.com.cn).

#### 2.9.2. Apoptosis Detection in Pancreatic Tissue Samples

Paraffin sections of pancreatic tissue were dewaxed and hydrated and then washed twice with xylene for 5 min. Tissue samples were then treated with different concentrations of ethanol (100, 95, 90, 80, and 70%) for 3 minutes and then treated with 1% Triton-100 and 3% H_2_O_2_-methanol solution for 15 min. Consequently, samples were treated with proteinase K at 37°C for 30 min. Streptavidin-FITC-labeled working solution was added to each section, and the reaction was incubated in a humidified chamber at 37°C for 1 h in the dark. Afterwards, each slice was dripped in prepared DAPI dye solution for 5 min at room temperature in the dark. Apoptotic cells were observed by fluorescence microscopy and imaged.

### 2.10. Protein Extraction and Western Blotting

#### 2.10.1. Protein Extraction

Whole cell and nuclear extracts were prepared for Western blot analysis. Control and experimental rats were fasted overnight for 8 h and then euthanized by intraperitoneal injection of 10% chloral hydrate (0.03 mL/kg). Islets were isolated from each SD rat pancreas according to previously described approach [11]. Samples were then washed twice with PBS and lysed in radioimmune precipitation assay buffer containing 50 mM Tris (pH 7.4), 150 mM NaCl, 1% Triton X-100, 1% sodium deoxycholate, and 0.1% SDS with protease inhibitors on ice for 30 min.

#### 2.10.2. Western Blotting

Protein concentration from cells and islet tissue was determined by bicinchoninic acid protein assays. Protein samples were separated by SDS-polyacrylamide gel and transferred to a polyvinylidene fluoride (PVDF) membrane (Sigma). Membranes were blocked with 5% fat-free milk in Tris-buffered saline (TBS) containing 0.1% Tween-20 and incubated overnight at 4°C with anti-TLR4 (1 : 1000, Abcam, UK, ab13867), anti-NF-*κ*B p65 (1 : 1000, Abcam, UK, ab16502), anti-GRP40 (1 : 200, Santa Cruz, sc-32905), anti-PLC (1 : 500, Abcam, UK, ab243181), anti-pAMPK*α*1 (1 : 1000, Abcam, UK, ab23875), anti-AMPK (1 : 500, Abcam, UK, ab3759), or anti-*β*-actin antibody (1 : 200, Abcam, UK, ab8226). Membranes were then incubated with horseradish peroxidase-labeled secondary antibody (1 : 500) for 1 hour at room temperature. Protein signals were visualized using the enhanced chemiluminescence detection system.

### 2.11. Immunofluorescence

Pancreatic paraffin sections from each group were dewaxed, hydrated, and pretreated using the heat-induced antigen retrieval technique. Each section was enzyme inactivated using 3% H_2_O_2_ in methanol for 10 min and blocked with ready-to-use goat serum for 20 min. The sections were then incubated with 1 : 50 diluted primary antibody (anti-insulin and anti-glucagon) incubated in a wet box for 2 hours at 37°C and 1 : 50 diluted second antibody FITC/TRITC incubated in the dark for 1 hour at 37°C. Each slice was then incubated in prepared DAPI dye solution for 5 min at room temperature in the dark and then sealed with antiextraction sealant. Cellular protein expression was observed under a fluorescence microscope, and three random regions were imaged.

### 2.12. Statistical Analysis

Results were expressed as mean ± S.E. Comparisons between groups were performed using ANOVAs. The LSD test was performed to compare the two groups. *P* < 0.05 was considered to be statistically significant.

## 3. Results

### 3.1. The Protective Effect of Metformin on Lipotoxicity-Induced Meta-Inflammation in *β*-Cells Is Regulated by the GPR40 Expression

**Figure 1:**
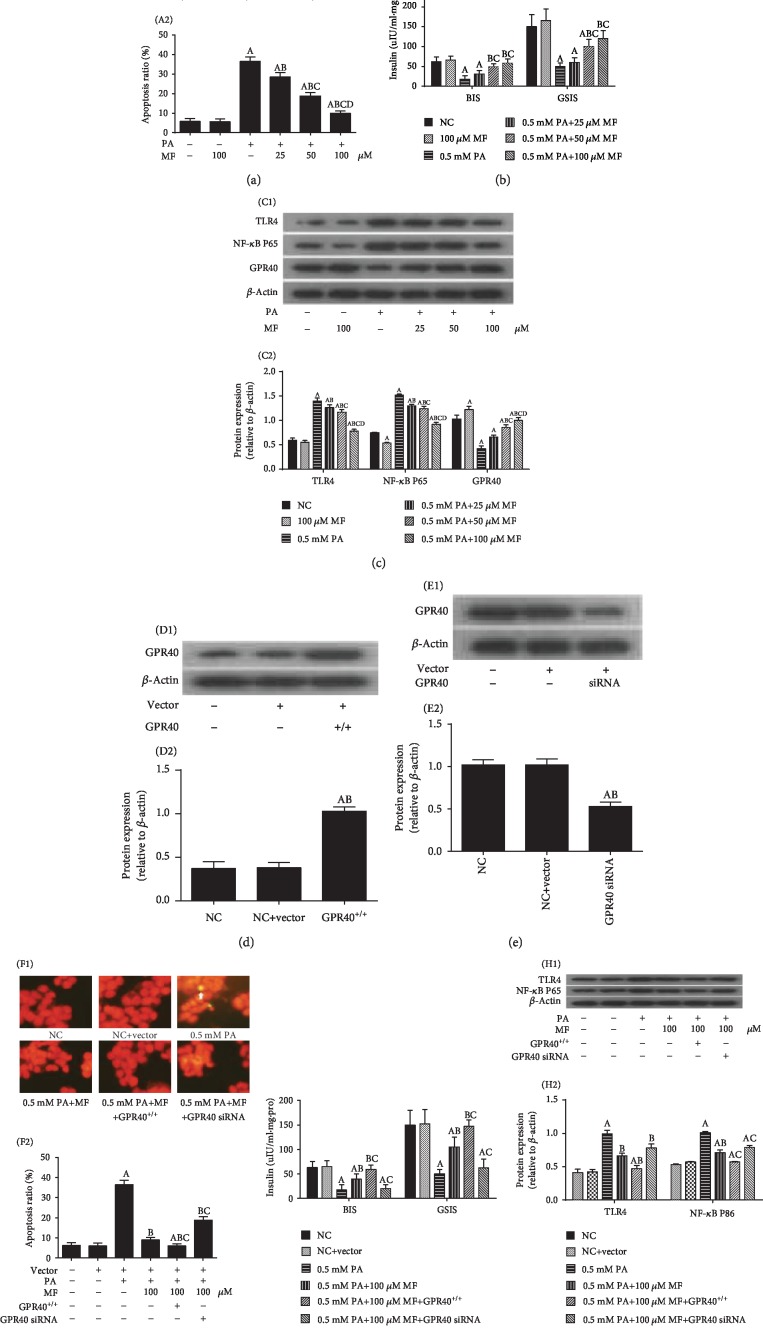
Adjustment GPR40 expression altered metformin's protective function on lipotoxicity in *β*-cells. All data is presented as mean ± SD of three independent experiments. (a) Metformin reduces PA-induced apoptosis in *β*-cells. (A1) Representative images from fluorescent microscopy in each group. The white arrow indicates apoptotic cells. (A2) Collective analyses of all three independent experiments. (b) Detection of basal insulin secretion (BIS) and glucose-stimulated insulin secretion (GSIS) by ELISA. (c) Expression of TLR4, NF-*κ*B subunit P65, and GPR40 detected by Western blotting. (C1) Representative Western blot images for each group. (C2) The ratio of target protein to *β*-actin. (d, e) GPR40 protein expression levels detected by Western blotting in GPR40-overexpressing transfected cells and siRNA transfected cells (siRNA). Regulation of GPR40 expression and the protective effects of metformin on PA -induced *β*-cell apoptosis (f), insulin secretion disorder (g), and TLR4 and NF-*κ*B subunit P65 protein expression (h). (a–c) ^A^*P* < 0.05 vs. NC group (without PA and MF), ^B^*P* < 0.05 vs. 0.5 mmol/L PA group, ^C^*P* < 0.05 vs. 0.5 mmol/L PA+25 *μ*mol/L MF group, and ^D^*P* < 0.05 vs. 0.5 mmol/L PA+50 *μ*mol/L MF group. (d, e) ^A^*P* < 0.05 vs. NC group, ^B^*P* < 0.05 vs. NC+vector group. (f–h) ^A^*P* < 0.05 vs. NC group (without PA and MF), ^B^*P* < 0.05 vs. 0.5 mmol/L PA group, and ^C^*P* < 0.05 vs. 0.5 mmol/L PA+100 *μ*mol/L MF group.

To observe the effect of metformin on *β*-cell lipotoxicity injury, lipotoxicity-injured *β*-cells were incubated with different concentrations of metformin, and TLR4 expression was assessed as a marker of meta-inflammation. We observed that metformin decreased apoptosis of *β*-cells (Figure 1(a)) in a concentration-dependent manner and increased insulin secretion, BIS, and GSIS (Figure 1(b)). Simultaneously, metformin concentration-dependently increased expression of GPR40 and decreased TLR4 and NF-*κ*B subunit P65 expression (Figure 1(c)).

We further validated whether GPR40 was involved in the protective effects of metformin using lentivirus-mediated overexpression (Figure 1(d)) or silencing of GPR40 in *β*-cells (Figure 1(e)). Our results showed that the upregulation of GPR40 improved the effect of metformin on lipotoxicity-induced apoptosis (Figure 1(f)), increased BIS and GSIS (Figure 1(g)), and reduced inflammation-related protein expression (Figure 1(h)). Conversely, the downregulation of GPR40 decreased the protective effects of metformin on *β*-cells (Figures 1(f)–1(h)).

### 3.2. GPR40 Was Involved in Metformin Reversing Metabolic Inflammation Key Marker (TLR4) Activation-Mediated *β*-Cell Injury

**Figure 2:**
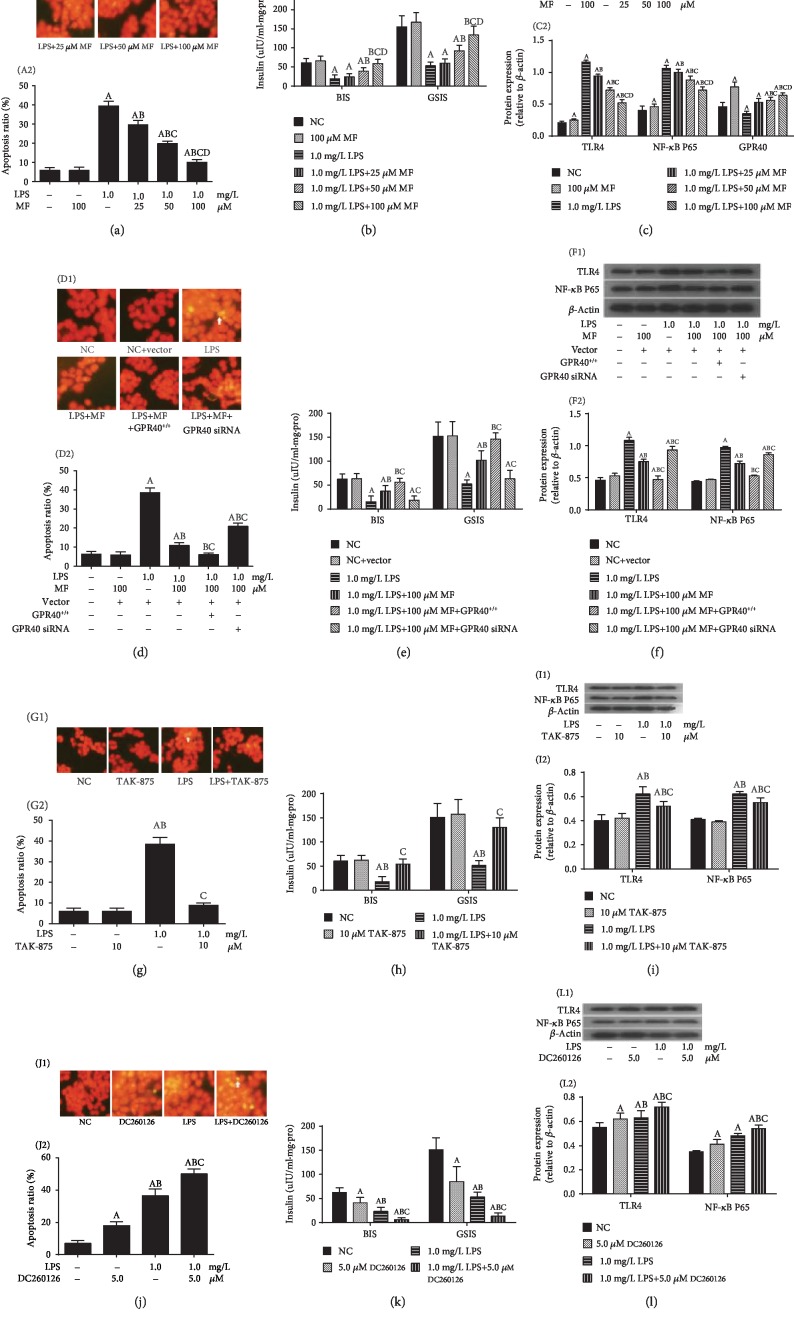
GPR40 and its involvement in metformin improves LPS-induced inflammatory injury. All data is presented as mean ± SD of three independent experiments. Metformin improves LPS-induced inflammatory apoptosis (a), insulin secretion disorder (b), and TLR4 and NF-*κ*B subunit P65 expression (c). Regulation of GPR40 expression and the protective effects of metformin on LPS-induced *β*-cell apoptosis (d), insulin secretion disorder (e), and TLR4 and NF-*κ*B subunit P65 expression (f). Activation of GPR40 by TAK-875 protects LPS-induced *β*-cell apoptosis (g), insulin secretion disorder (h), and TLR4 and NF-*κ*B subunit P65 expression (i). Inhibition of GPR40 by DC260126 aggravated LPS-induced apoptosis (j), insulin secretion disorder (k), and TLR4 and NF-*κ*B subunit P65 expression (l). (a–c) ^A^*P* < 0.05 vs. NC group (without LPS and MF), ^B^*P* < 0.05 vs. 1.0 mg/L LPS group, ^C^*P* < 0.05 vs. 1.0 mg/L LPS+25 *μ*mol/L MF group, and ^D^*P* < 0.05 vs. 1.0 mg/L LPS+50 *μ*mol/L MF group. (d–f) ^A^*P* < 0.05 vs. NC group (without LPS and MF), ^B^*P* < 0.05 vs. 1.0 mg/L LPS group, and ^C^*P* < 0.05 vs. 1.0 mg/L LPS+100 *μ*mol/L MF group. (g–i) ^A^*P* < 0.05 vs. NC group (without LPS and TAK-875), ^B^*P* < 0.05 vs. TAK-875 group, and ^C^*P* < 0.05 vs. LPS group. (j–l) ^A^*P* < 0.05 vs. NC group (without LPS and DC260126), ^B^*P* < 0.05 vs. DC260126 group, and ^C^*P* < 0.05 vs. LPS group.

Next, we investigated the specificity of the relationship between protective effects of metformin and metabolic inflammation key marker TLR4. We used LPS, a specific agonist of TLR4, to induce *β*-cell inflammatory injury and then treated cells with metformin. We found that metformin improved LPS-induced inflammatory apoptosis in *β*-cells (Figure 2(a)), increased insulin secretion (Figure 2(b)) and GPR40 expression, and inhibited the expression of TLR4 and NF-*κ*B subunit P65 (Figure 2(c)) in a concentration-dependent manner.

We then used lentivirus-mediated silencing or overexpression of GPR40 to verify the effect of GPR40 expression on metformin's protective role in LPS-injured *β*-cells. Our results demonstrated that the upregulation of GPR40 improved the effect of metformin on LPS-induced apoptosis (Figure 2(d)), increased BIS and GSIS (Figure 2(e)), and reduced inflammation-related protein expression (Figure 2(f)). Conversely, the downregulation of GPR40 attenuated the protective effect of metformin on *β*-cells (Figures 2(d)–2(f)).

To further confirm the effect of GPR40 on *β*-cell inflammatory injury, we treated cells with GPR40 agonists or inhibitors after LPS-induced *β*-cell inflammatory injury. Activation of GPR40 improved lipotoxic-induced apoptosis (Figure 2(g)), increased BIS and GSIS (Figure 2(h)), and decreased expression of TLR4 and NF-*κ*B subunit P65 (Figure 2(i)); and inhibition of GPR40 activity significantly aggravated the inflammatory injury of LPS on *β*-cells (Figures 2(j)–2(l)).

### 3.3. Downstream Target of GPR40 (PLC-IP3) Is Involved in the Protective Effect of Metformin on Lipotoxicity Inflammatory *β*-Cells

**Figure 3:**
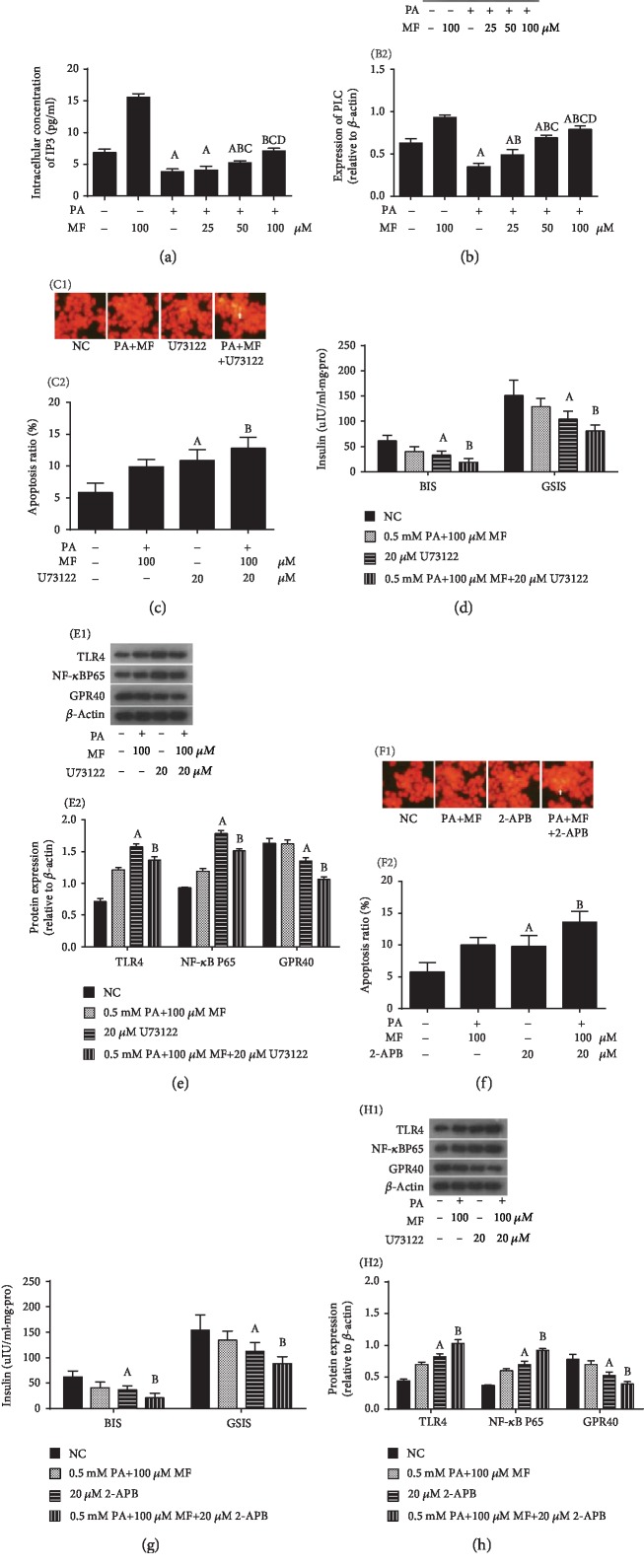
The relationship between PLC-IP3 and the protective effects of metformin on lipotoxicity inflammatory injury. Results shown are representative of at least 3 experiments. Metformin concentration-dependently increased the expression of PLC (a) and IP3 (b) in *β*-cells. (c–h) Inhibition of PLC or IP3 expression attenuated the protective effects of metformin on lipotoxicity inflammatory injury. (c, f) The rate of apoptosis in *β*-cells. (d, g) Levels of BIS and GSIS. (e, h) Expression levels of TLR4, NF-*κ*B subunit P65, and GPR40. (a, b) ^A^*P* < 0.05 vs. NC group (without PA and MF), ^B^*P* < 0.05 vs. 0.5 mmol/L PA group, ^C^*P* < 0.05 vs. 0.5 mmol/L PA+25 *μ*mol/L MF group, and ^D^*P* < 0.05 vs. 0.5 mmol/L PA+50 *μ*mol/L MF group. (c–h) ^A^*P* < 0.05 vs. NC group (without PA and MF), ^B^*P* < 0.05 vs. 0.5 mmol/L PA+100 *μ*mol/L MF group.

We then investigated the possible association of GPR40 downstream signaling (PLC-IP3) with metformin treatment. We first observed the effect of metformin on PLC-IP3 and then used PLC-IP3 specific inhibitors to interfere with the effects of metformin on lipotoxicity in *β*-cells. Our results showed that metformin concentration-dependently increased the level of IP3 in *β*-cells (Figure 3(a)) and PLC (Figure 3(b)) expression. Inhibition of PLC or IP3 reduced the protective effect of metformin on lipotoxicity inflammatory *β*-cells, which was characterized by increased apoptosis of lipotoxicity inflammatory *β*-cells (Figures 3(c) and 3(f)), decreased insulin secretion (Figures 3(d) and 3(g)), reduced expression of GPR40, and increased expression of TLR4 and NF-*κ*B subunit P65 (Figures 3(e) and 3(h)).

### 3.4. The Protective Effect of Metformin on Lipotoxicity Was Partially Regulated by AMPK Activity

**Figure 4:**
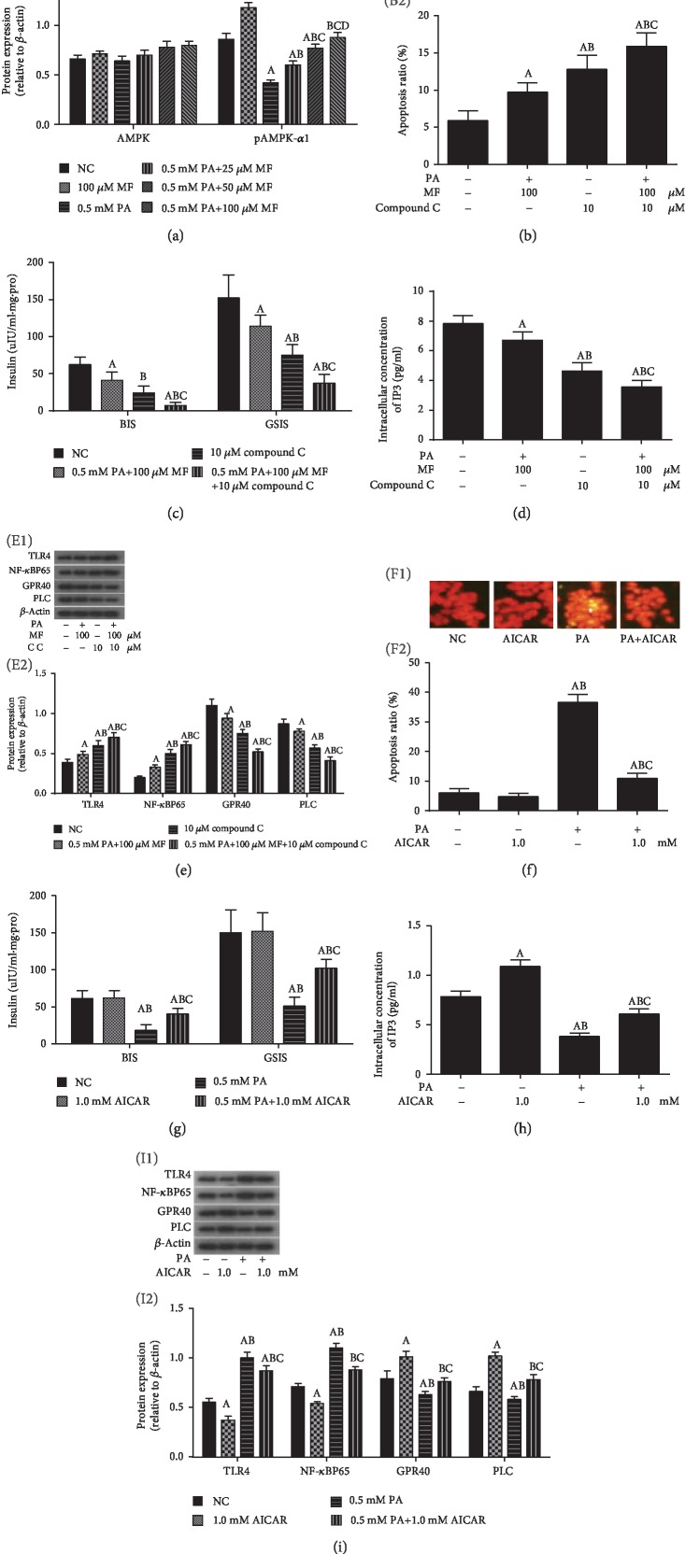
The protective effect of metformin was partially independent of AMPK activity. (a) Metformin concentration-dependently increased the expression of AMPK. (A1) Representative Western blot images for each group. (A2) The ratio of target protein to *β*-actin. (b–e) The relationship between metformin-regulated lipotoxicity and AMPK inhibitor. (b) The rate of apoptosis in *β*-cells. (c) Levels of BIS and GSIS. (d) Expression of IP3 in *β*-cells. (e) Expression levels of TLR4, NF-*κ*B subunit P65, and GPR40. (f–i) Effect of AICAR on PA-induced *β*-cell injury. (f) The rate of apoptosis in *β*-cells. (g) Levels of BIS and GSIS. (h) Expression of IP3 in *β*-cells. (i) Expression levels of TLR4, NF-*κ*B subunit P65, and GPR40. (a) ^A^*P* < 0.05 vs. NC group (without PA and MF), ^B^*P* < 0.05 vs. 0.5 mmol/L PA group, ^C^*P* < 0.05 vs. 0.5 mmol/L PA+25 *μ*mol/L MF group, and ^D^*P* < 0.05 vs. 0.5 mmol/L PA+50 *μ*mol/L MF group. (b–e) ^A^*P* < 0.05 vs. NC group, ^B^*P* < 0.05 vs. 0.5 mmol/L PA+100 *μ*mol/L MF group, and ^C^*P* < 0.05 vs. 10 *μ*mol/L compound C group. (f–i) ^A^*P* < 0.05 vs. NC group, ^B^*P* < 0.05 vs. 1.0 mmol/L AICAR group, and ^C^*P* < 0.05 vs. 0.5 mmol/L PA+1.0 mmol/L AICAR group.

First, we observed the effect of metformin on AMPK activity in lipotoxicity *β*-cells and found that metformin can increase the activation of AMPK in a concentration-dependent manner (Figure 4(a)). Consequently, we investigated whether the AMPK inhibitor compound C may interfere with the effect of metformin on lipotoxicity. Our data suggested that the inhibition of AMPK activity partially reduced the protective effect of metformin (Figures 4(b)–4(e)). Furthermore, we examined the effect of AMPK specific activator AICAR on lipotoxicity *β*-cells. We found that AICAR inhibits *β*-cell apoptosis (Figure 4(f)), improves insulin secretion (Figure 4(g)), increases GPR40-IP3-PLC expression, and inhibits TLR4 and NF-*κ*B subunit P65 expression (Figures 4(h) and 4(i)).

### 3.5. Effect of Metformin on High-Fat Diet-Induced Inflammatory Injury in Obese Rats

**Figure 5:**
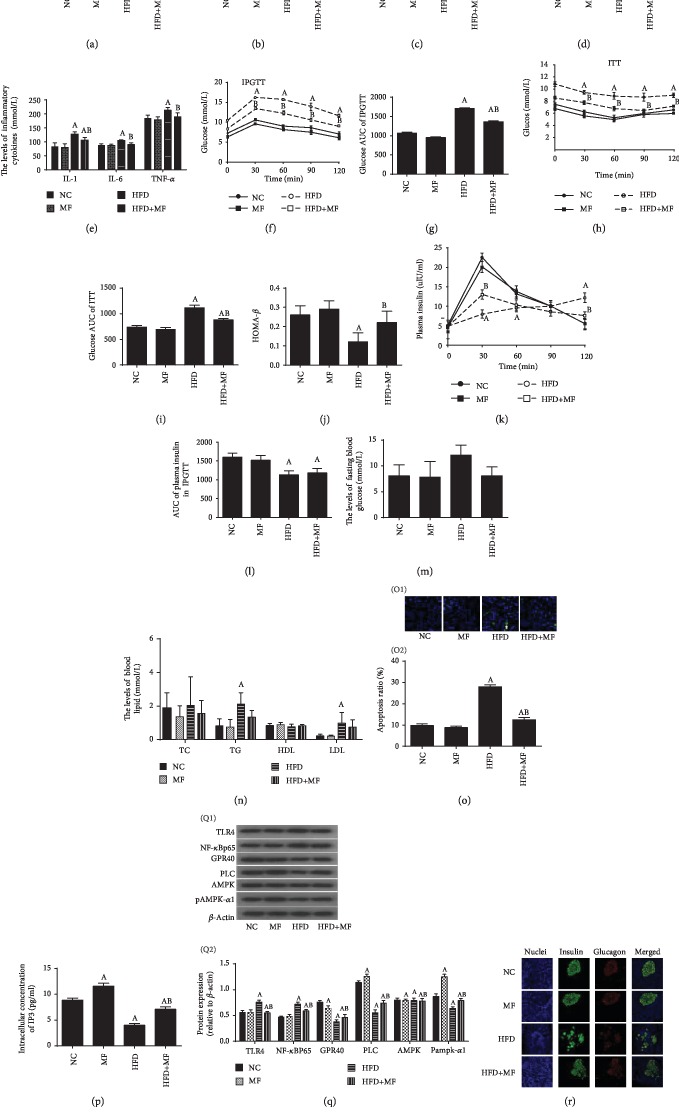
Protective effects of metformin on high-fat diet-induced inflammatory injury in obese rats. (a) The average weight of each treatment group. (b) Lee's index. (c) HOMA-IR. (d) Free fatty acid A (FFA). (e) Metformin decreases the levels of IL-1, IL-6, and TNF-*α*. (f) IPGTT. (g) Glucose AUC of IPGTT. (h) ITT. (i) Glucose AUC of ITT for each group. (j) HOMA-*β*. (k) Plasma insulin levels during IPGTT. (l) The AUC for plasma insulin concentration during IPGTT. (m) Fasting blood glucose levels. (n) Blood lipid levels for each group: total cholesterol (TC), triglyceride (TG), high-density lipoprotein (HDL), and low-density lipoprotein (LDL). (o) Metformin reduces pancreatic cell apoptosis induced by high-fat diet. (O1) Representative images from fluorescent microscopy for each group. The white arrows indicate apoptotic cells. (O2) Collective analyses of all three independent experiments. (p) Metformin increases the levels of IP3. (q) Expression of TLR4, NF-*κ*B subunit P65, GPR40-PLC, AMPK, and pAMPK-*α*1 detected by Western blot. (Q1) Representative Western blot images for each group. (Q2) The ratio of target protein to *β*-actin. (r) Representative images of immunofluorescence from pancreatic tissue for each group. ^A^*P* < 0.05 vs. NC group, ^B^*P* < 0.05 vs. HFD group.

Diet-induced obese SD rats were used to verify our findings in vitro. The results revealed that metformin reduces body weight (Figure 5(a)), Lee's index (Figure 5(b)), HOMA-IR (Figure 5(c)), FFA (Figure 5(d)), TNF-*α*, IL-1, and IL-6 (Figure 5(e)) levels, and the AUC of glucose IPGTT (Figure 5(g)) and ITT (Figure 5(i)) and increases the HOMA-B (Figure 5(j)) and the AUC for plasma insulin concentration during IPGTT (Figure 5(l)) in rats fed with HFD. However, there was no significant difference in fasting blood glucose (Figure 5(m)), total cholesterol, triglyceride, high-density lipoprotein, and low-density lipoprotein levels (Figure 5(n)) between the obese group and the metformin intervention obese group. In addition, metformin decreased the pancreatic cell apoptosis induced by HFD (Figure 5(o)), increased the levels of IP3 (Figure 5(p)) and the expression of GPR40, PLC, and pAMPK*α*1, inhibited the expression of TLR4 and NF-*κ*B subunit P65 (Figure 5(q)), and improved the distribution and quantity of *α*-cells and *β*-cells in the pancreas (Figure 5(r)).

## 4. Discussion

In this study, we found that metformin could reduce the lipotoxicity-induced insulin secretion deficiency in *β*-cells, decrease cell apoptosis, and inhibit the activation of metabolic inflammation key marker (TLR4-NF-*κ*B). In addition, we discovered that the GPR40 expression alters metformin's protective function on lipotoxicity *β*-cells. We also found that GPR40 affected metformin's role in inhibiting activated TLR4-induced *β*-cell injury. Furthermore, downstream signaling protein PLC-IP3 of GPR40 was involved in the protective effect of metformin on meta-inflammation, and the above process of metformin was partially regulated by AMPK activity. We also observed that metformin could reduce body weight and inflammatory-related factors, improve *β*-cell function, increase GPR40-PLC-IP3 expression, and inhibit activation of the TLR4-NF-*κ*B pathway in an obese rat model.

The present study showed that metformin has a direct protective effect on lipotoxicity *β*-cells. Yet, the administration of metformin alone did not cause a decrease in cells or an increase in insulin secretion. This phenomenon is consistent with the clinic findings, which indicated that metformin alone does not increase the risk of hypoglycemia in diabetic patients [34]. Hence, metformin could be administered to nondiabetic patients, such as patients with fatty liver [35] and polycystic ovary syndrome (PCOS) [36], without causing hypoglycemia. Moreover, clinical studies have suggested that metformin has a protective effect on *β*-cells, independent of its hypoglycemic effect [37]. These data suggest that metformin has an additional protective effect on *β*-cells; however, the specific mechanisms need to be investigated [38, 39].

Furthermore, we examined whether GPR40 was involved in metformin's lipotoxicity protection. Our results demonstrated that high expression of GPR40 was directly associated with the protective effect induced by metformin, while its inhibition reduced the effects of metformin. So far, a number of studies, including our previous work, have confirmed the benign intervention of GPR40 on *β*-cells [11, 40] and the protective effect of GPR40 on islet *β*-cells [41]. In addition, decreased GPR40 expression was found in diabetic patients [42]. The above evidence indicates that the upregulation and activation of GPR40 have a protective role on *β*-cells, which is consistent with our findings. Moreover, studies have suggested that PA-induced TLR4-NF-*κ*B activation in *β*-cells leads to inflammatory apoptosis and insulin secretion deficiency [24, 43]. However, whether the positive-mediated effect of GPR40 on metformin is related to the inhibition of TLR4-NF-*κ*B activity still remains unclear and needs to be further investigated.

In order to confirm whether GPR40 mediates the effect of metformin through the inhibition TLR4-NF-*κ*B activity, we used LPS as a specific agonist for TLR4 to induce *β*-cell damage. We found that GPR40 was involved in metformin reversing LPS-induced *β*-cell inflammatory injury. From the above experiments, we confirmed that GPR40 could inhibit LPS-induced or lipotoxicity-induced TLR4-NF-*κ*B activation by metformin. It has been demonstrated that GPR40 has anti-inflammatory actions [44], thus supporting our conclusion.

The exact mechanisms through which GPR40 mediates metformin-regulated *β*-cell lipotoxicity inflammatory injury still remain unclear. PLC-IP3 is a downstream signaling pathway of GPR40, which regulates *β*-cell insulin secretion [45]. Our previous study has shown that PLC is involved in GPR40-mediated pioglitazone antagonism of lipotoxic *β*-cell apoptosis and oxidative stress [24]. Several other studies have also shown that PLC-IP3 is involved in GPR40-mediated DHA-induced neuronal differentiation and neurite outgrowth in adult rat stem cells [46]. In this study, we further verified that PLC-IP3 is involved in the mediating effects of GPR40 on metformin. There are several publications supporting our findings; existing studies have suggested that palmitate-induced ER Ca (2+) depletion by PLC-IP3R signaling upregulates ER stress proteins and that mitochondrial dysfunction leads to beta cell dysfunction. In addition, studies have reported that metformin has protective effects on palmitic acid-induced ER-mitochondrial dysfunction [47]. Another study performed in breast cancer patients found that metformin inhibited the activity of PLC [48], which is inconsistent with our findings. We hypothesized that the possible reason was the cell-specific or bidirectional effects of metformin on cells; metformin may inhibit tumor cell growth by inhibiting the activity of PLC. In our study, metformin alleviated *β*-cell apoptosis induced by lipotoxicity via PLC.

Next, we investigated whether the GPR40-IP3-PLC pathway was mediated or affected by the AMPK activation. Our results indicated that metformin could activate AMPK phosphorylation in lipotoxicity *β*-cells. We further found that the activation of AMPK can antagonize the damage to lipotoxicity, while its inhibition can partially decrease the protective effect of metformin. The above results suggest that the effect of metformin on lipotoxicity is partially dependent on AMPK activity. In addition, recent studies have confirmed that metformin could suppress the formation of fat by regulating the activity of Runx2 and PPAR*γ* [49]. This effect appears to be independent of the activity of AMPK.

Moreover, we further confirmed the protective effect of metformin *in vivo.* Metformin reduced body weight, decreased the levels of inflammatory factors, improved islet cell function, increased the expression of GPR40-PLC-IP3 and pAMPK*α*1, and inhibited the activation of TLR4-NF-*κ*B in HFD-induced obese SD rats. In order to further explain the effect of metformin on islet beta-cell function, we used statistics to control the effects of weight and insulin resistance, and we analyzed the effect of metformin on islet *β*-cells. The results showed that the protective effect of metformin on islet cell function was still present (*P* < 0.05, see supplementary materials [Supplementary-material supplementary-material-1]). Our results suggested that metformin could improve *β*-cell function. Other studies have also reported the direct improvement of metformin in animal and human *β*-cells [50, 51], which further supported our experimental conclusions. However, metformin administration in normal SD rats did not affect the animal body weight or insulin secretion, which is consistent with clinical studies suggesting that metformin has self-limiting effects to reduce body weight [52]. Previous experiments have also demonstrated that metformin improves the metabolism of obese rats [53]. Our results showed that the high-fat diet caused a decrease in fasting insulin concentration in obese rats, which may be related to longer high-fat diet intervention time. Studies have suggested that the intervention time of high-fat diet is 4-8 weeks [54, 55]. And in the experiment, the high-fat diet lasted 16 weeks. Thus, we speculated that the metabolic stress induced by prolonged high-fat diet gradually leads to a decrease in islet function from decompensation to decompensation.

To sum up, we found that metformin could inhibit lipotoxicity-induced meta-inflammation damage in *β*-cells by regulating the GPR40-PLC-IP3 signaling pathway. Nevertheless, this study has certain limitations. Firstly, we did not investigate the mechanism of metformin acting on GPR40. Secondly, we used mouse islet cell line, which is different from islet *β*-cells found in humans. Third, our research lacked some negative control group, so our conclusions needed to be verified by subsequent experiments.

## Data Availability

The data used to support the findings of this study are included within the article.

## Abbreviations

AECs: Alveolar epithelial cells
AICAR: 5-Aminoimidazole-4-carboxamide1-*β*-D-ribofuranoside
AMPK: AMP-activated protein kinase
ANOVAs: Analyses of variance
BIS: Basal insulin secretion
DAPI: 4,6-Diamidino-2-phenylindole
DHA: Docosahexaenoic acid
DMEM: Dulbecco's modified Eagle's medium
DMSO: Dimethylsulfoxide
FBS: Fetal bovine serum
FFA: Free fatty acid A
GPR40: G protein-coupled receptor 40
GSIS: Glucose-stimulated insulin secretion
HbA1c: Glycosylated hemoglobin
HDL-C: High-density lipoprotein cholesterol
HEPES: Hydroxyethyl piperazine ethanesulfonic acid
HSFD: High-sugar, high-fat diet
HOMA-B: Homeostasis model assessment of beta cell function
HOMA-IR: Homeostasis model assessment to quantify insulin resistance
HOMA-S: Homeostasis model assessment of insulin sensitivity
IL-1: Interleukin-1
IL-6: Interleukin-6
IP3: Inositol 1, 4, 5-trisphosphate
IPGTT: Intraperitoneal glucose tolerance test
ITT: Insulin tolerance test
JNK: c-Jun N-terminal protein kinase
LDL-C: Low-density lipoprotein cholesterol
LPS: Lipopolysaccharide
MF: Metformin
MyD88: Myeloid differentiation factor 88
NC group: Normal control group
NF-*κ*B: Nuclear factor-kappa B
NLR: Neutrophil to lymphocyte ratio
OD value: Optical density value
PA: Palmitic acid
PBS: Phosphate buffer saline
PCOS: Polycystic ovary syndrome
PLC: Phospholipase C
PVDF: Polyvinylidene fluoride
siRNA: Small interfering RNA
TBS: Tris-buffered saline
TC: Total cholesterol
TG: Triglycerides
TLR4: Toll-like receptor 4
TNF-*α*: Tumor necrosis factor-*α*
TUNEL: Terminal-deoxynucleoitidyl transferase-mediated nick end labeling.

## Supplementary Materials

Supplementary Materials Metformin improved beta cell function independent of weight and insulin resistance.
