# Supplementary material for: Metformin Reduces Lipotoxicity-Induced Meta-Inflammation in β-Cells through the Activation of GPR40-PLC-IP3 Pathway
Source: J Diabetes Res. 2019 Dec 18;2019:7602427. doi: 10.1155/2019/7602427 (PMC6948338; doi:10.1155/2019/7602427)
Supplement: Supplementary Materials — Metformin improved beta cell function independent of weight and insulin resistance. [file 7602427.f1.pdf]

Supplementary Material 1

Metformin improved beta cell function independent of weight and insulin resistance

In order to further explain the effect of metformin on islet beta-cell function, we used covariance analysis to control the effects of weight and insulin resistance, and we analyzed the effect of metformin on islet β cells function. As shown in Table 1, after controlling weight and insulin resistance, the AUC of IPGTT, AUC of ITT and the poptosis of pancreatic cell in the MF+HFD group were lower than those of the HFD group, while the HOMA-β was elevated ($P < 0.05$).

Table 1 - Effect of metformin on high fat diet induced islet dysfunction in obese rats

	NC	MF	HFD	MF+HFD	P value
**Fasting blood-glucose (mmol/L)**	8.0±2.2	7.9±3	11.98±2.1	8.1±1.6	
**Fasting blood-insulin (μIU/ml)**	4.94±0.81	4.70±1.0	6.18±0.83	4.84±1.1	
**AUC of IPGTT**	1056±38.38	936±38.21	1707±37.56 [a]	1349±33.07 [ab]	<0.05
**AUC of ITT**	738.6±27.92	684±30.86	1110±47.36 [a]	870±27.5 [ab]	<0.05
**HOMA-β**	0.26±0.05	0.29±0.046	0.12±0.049 [a]	0.22±0.059 [ab]	<0.05
**Apoptosis (%)**	9.8±0.8	8.8±0.7	28±0.9 [a]	12.5±1 [ab]	<0.05

[a]$p < 0.05$ vs.NC group , [b]$P < 0.05$ vs. HFD group.
